# Identification of manganese efficiency candidate genes in winter barley (*Hordeum vulgare*) using genome wide association mapping

**DOI:** 10.1186/s12864-016-3129-9

**Published:** 2016-10-04

**Authors:** Florian Leplat, Pai Rosager Pedas, Søren Kjærsgaard Rasmussen, Søren Husted

**Affiliations:** Department of Plant and Environmental Sciences, University of Copenhagen, Thorvaldsensvej 40, 1871 Frederiksberg, Copenhagen Denmark

**Keywords:** Chlorophyll (Chl) *a* fluorescence, Genome-wide association (GWA), Germin-like protein (GLP), Inductively coupled plasma - optical emission spectrometry (ICP-OES), Manganese (Mn) efficiency, Mn-SOD, Oxalate-oxidase (OxO), Photosystem II (PSII)

## Abstract

**Background:**

Manganese (Mn) has several essential functions in plants, including a role as cofactor in the oxygen evolving complex (OEC) of photosystem II (PSII). Manganese deficiency is a major plant nutritional disorder in winter cereals resulting in significant yield reductions and winter kill in more severe cases. Among the winter cereals, genotypes of winter barley are known to differ considerably in tolerance to Mn deficiency, but the genes controlling the Mn deficiency trait remains elusive.

**Results:**

Experiments were conducted using 248 barley varieties, cultivated in six distinct environments prone to induce Mn deficiency. High-throughput phenotyping for Mn deficiency was performed by chlorophyll *a* (Chl *a*) fluorescence analysis to quantify the quantum yield efficiency of PSII. High-throughput phenotyping in combination with ICP-OES based multi-element analyses allowed detection of marker-trait associations by genome wide association (GWA) mapping. Several key candidate genes were identified, including PSII subunit proteins, germin like proteins and Mn superoxide dismutase. The putative roles of the encoded proteins in Mn dependent metabolic processes are discussed.

**Conclusions:**

Fifty-four candidate genes were identified by Chl *a* fluorescence phenotyping and association genetics. Tolerance of plants to Mn deficiency, which is referred to as Mn efficiency, appeared to be a complex trait involving many genes. Moreover, the trait appeared to be highly dependent on the environmental conditions in field. This study provides the basis for an improved understanding of the parameters influencing Mn efficiency and is valuable in future plant breeding aiming at producing new varieties with improved tolerance to cultivation in soil prone to induce Mn deficiency.

**Electronic supplementary material:**

The online version of this article (doi:10.1186/s12864-016-3129-9) contains supplementary material, which is available to authorized users.

## Background

Deficiency of the essential micronutrient manganese (Mn) remains an unsolved problem that has a severe impact on crop production worldwide [1–4]. In addition to substantial yield losses, suboptimal use of nitrogen, phosphorus and water are marked side-effects of Mn deficiency. It is prevalent in areas with well aerated and high pH soils containing free carbonates and with high organic matter content [5]. Manganese deficiency often occurs as a latent disorder with no visual symptoms making it difficult to diagnose and follow up with timely Mn remediation [6]. Mn deficient plants have a decreased lignin content [7] and are therefore more prone to be infected by pathogens [8, 9] and have marked decreased winter hardiness [6, 10]. Application of soluble Mn-fertilizers to the soil is an ineffective way to correct Mn-deficiency, as the added Mn is instantaneously made unavailable by oxidation to MnO_2_, due to soil chemical conditions [5]. Foliar applications of soluble manganous sulphate are more effective, but this is time consuming, expensive and often impractical for farmers cultivating marginal lands [11]. A second way for farmers to fight Mn deficiency induced yield losses is by deploying plant varieties with an improved tolerance to growth in soils with low Mn availability, defined as Mn efficient varieties [3, 12]. Improving nutrient efficiencies by exploiting genetic diversity in plants and strategies to implement such traits into crop breeding have previously been suggested to improve plant productivity [13–16].

Previous studies on barley varieties have identified various phenotypes in terms of tolerance to low Mn availability in soil [3, 17], implying a genetic control of the trait. However, the genetic mechanisms involved in the ability of plants to cope with low amounts of plant-available Mn have not yet been clarified. Several physiological mechanisms have been suggested to be involved in Mn efficiency in barley. For instance, it has been shown in barley that the Mn efficient variety Vanessa has a four-fold higher Mn uptake capacity compared with the Mn inefficient variety Antonia when exposed to sub-nanomolar Mn concentrations [18]. A follow-up study has suggested that this difference in uptake capacity is caused by different expression levels of the Mn transporter HvIRT1 [19]. However, Mn uptake and acropetal translocation involve many different transport pathways and their role in controlling Mn efficiency remains unknown [20]. It has also been proposed that chloroplasts are a main target for Mn deficiency and that Mn efficiency in barley is significantly influenced by processes linked to the stability and photochemical efficiency of the photosynthetic apparatus [21]. The photosynthetic apparatus seems more unstable when exposed to Mn limitations, with the amount of PsbA (D1) protein being reduced in the Mn inefficient variety Antonia compared to the Mn efficient variety Vanessa [22]. In addition, the ability to perform state transitions is only significantly decreased in the Mn inefficient varieties [21]. Furthermore, it has recently been suggested that exudation of enzymes to the rhizosphere (phytases dissolving organic chelated Mn) is involved in the superior Mn efficiency of certain ancient barley landraces [23].

Manganese activates more than 35 enzymes in plant metabolism, including processes such as the activation of the enzyme phenylalanine ammonia-lyase (PAL) in the shikimic pathway [7], Golgi localized glycosyl transferases [24, 25], decarboxylases and dehydrogenases in the tricarboxylic acid cycle [5]. A few enzymes and processes have an irreplaceable requirement for Mn in plants: i) oxalate oxidase (OxO) resulting in hydrogen peroxide production involved in pathogen attack [26]; ii) Mn superoxide dismutase (Mn-SOD) as a key enzyme involved in scavenging of reactive oxygen species in mitochondria and peroxisomes [27, 28]; iii) the oxidation of water (Hill-reaction) occurring in the oxygen-evolving complex (OEC) of photosystem II (PSII). Chl *a* fluorescence measurements to diagnose Mn deficiency have proven to be a powerful research tool for measuring the severity of Mn deficiency and the impact of different Mn fertilizers [6, 10]. Chl *a* fluorescence is an *in-vivo* non-invasive and non-destructive technique enabling quantification of photosynthetic efficiency such as quantum yield efficiency of PSII (F_V_/F_M_) [29]. This method can be extended to a high-throughput phenotyping method allowing for screening a large collection of cultivars.

In addition to phenotyping methods, the tremendous progresses made in genotyping major crop plants have enabled the search for QTLs and the identification of the underlying genes [30, 31]. However, very little work has so far been undertaken on the genetic dissection of the quantitative traits controlling the adaptive response of crops to abiotic stress, including micronutrient deficiencies. QTL mapping and GWA have previously been shown to be promising methods to identify and characterize loci for nutrient efficiency [13, 32–35]. Initial genetic studies using yield improvements in response to Mn fertilization as a quantitative trait have suggested that Mn efficiency might be controlled by a single locus [36, 37]. Several RFLP markers have been identified on barley chromosome 4HS linked to the *Mel1* locus for Mn efficiency. The role of *Mel1* has been confirmed in field trials and glasshouse experiments and included in breeding programs in South Australia [38].

Barley is the fourth most important cereal crop in the world [39] and it is a widely geographically adapted and stress tolerant crop. In addition, co-localization of barley genetic loci within other cereal species (synteny) is available [40, 41], and the major progress achieved by sequencing the barley genome [42, 43] and its diploid nature, makes barley a versatile crop for genetic studies. Together with high-throughput genotyping single nucleotide polymorphism (SNP) platforms combined with fast phenotyping methods, GWA is a suitable tool to perform genetic studies of many traits and identified new genes.

The objectives of the current study were: to induce Mn deficiency in a collection of 248 European winter barley varieties cultivated under field and greenhouse conditions, to determine the variation in Mn efficiency by Chl *a* fluorescence and leaf tissue Mn concentrations by ICP-OES, and subsequently to carry out a GWA in order to provide a set of SNPs associated with the trait, followed by the identification of candidate genes involved in Mn efficiency.

## Methods

### Experimental design

#### Plant material

The population consisted of 248 winter barley accessions (*Hordeum Vulgare*) collected across Europe (Additional file 1). The countries of origin most represented by the population were Denmark, France, Germany, Italy and the United Kingdom. A first set of 111 varieties came directly from the ExBarDiv collection (Genomics-assisted analysis and exploitation of barley diversity, ERA-PG funded project), with commercial cultivars released over the last 60 years. The collection was supplemented by 18 double-haploid lines from Sejet Plant Breeding (Denmark) and 27 varieties from CREA (Italy). Finally 92 varieties from European plant breeding companies were added. The population contained 139 two-row type and 109 six-row type varieties.

#### Greenhouse experiment

Plants were cultivated under greenhouse conditions in the autumn 2013. In order to induce Mn deficient conditions, soil was collected from Sweden (55.58° N, 14.05° E, Skåne area) that is known to induce Mn deficiency in winter crops (unpublished data). Mn deficiency has been observed in uncompact and loose soil, therefore the soil was mixed with fine and coarse Leca® (Light Expanded Clay Aggregate) and perlite. In terms of volume, the proportions were 45/25/15/15 % respectively of soil, perlite, fine Leca® (2–6 mm) and coarse Leca® (6–10 mm). Natural light was used and temperatures were maintained between 7 and 13 °C. The whole collection was sown in 2 L pots containing three plants of the same variety. A randomized complete block design was used with four replicates of each variety. Pots were watered every two weeks directly from the bottom of the pots by capillarity for a period of 10 min before the water was removed.

#### Field experiments

Experiments were carried out in 2011, 2012 and 2013 (Table 1). In September 2011, one field previously known to induce severe Mn deficiency was sown in Sweden (55.94° N, 14.19° E, Skåne area). In 2012 and 2013, two fields representing prevalent Danish sandy soil types were sown in Denmark (Saunte: 56.04° N, 12.30° E; Lejre: 55.38° N, 11.57° E; both sites at Sealand), where Mn deficiency had previously been observed. Field plots consisted of replicates of one meter long lines representing one variety. Replicated randomized designs were applied.

**Table 1:** Summary of the experiment framework used for Mn efficiency screening

Environment	Sowing	Screening date	Abbreviation	Population size	Number of replicates	Growth stage	Phenotyping	Number of observations
Kristianstad	Sep-11	Nov-11	KS11	112	10	5 leaves to beginning of tillering	Chl *a*	3840
Lejre	Sep-12	May-13	LJ12	233	2	End of tillering	Chl *a*	1728
Saunte	Sep-12	May-13	ST12	233	2	End of tillering	Chl *a*	1512
Lejre	Sep-13	Nov-13	LJ13	248	3	Beginning of tillering	Chl *a*	2376
Greenhouse	Nov-13	Dec-13	GH13A	248	2	3-4 leaves unfolded	Chl *a* ICP-OES	1584
Greenhouse	Nov-13	Jan-14	GH13B	248	2	5 leaves to beginning of tillering	Chl *a*	1584

#### Chl a fluorescence phenotyping

**Fig. 1:**
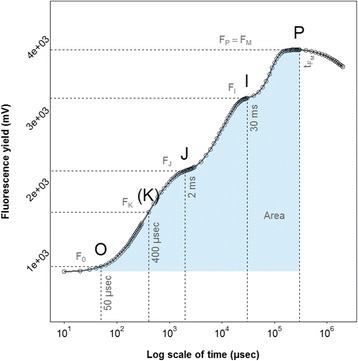
A typical Chl a fluorescence transient from a barley plant grown at optimal conditions. The transient is plotted on a logarithmic time scale from 50 μs to 1 s. The features that give rise to the O-J-I-P designation are highlighted. The marks (dashed lines) refer to the selected fluorescence steps used for the calculation of structural and functional parameters. The signals are: the fluorescence intensity F_0_ (at 50 μs), the fluorescence intensities F_J_ (at 2 ms) and F_I_ (at 30 ms) and the maximum fluorescence intensity F_P_ = F_M_ (at tF_M_). The blue shade under the curve represents the area

Chl *a* fluorescence was used to screen all experiments for Mn efficiency. Field and greenhouse fluorescence measurements were conducted using a handheld Plant Efficiency Analyser device (Handy PEA, Hansatech Instruments Limited, King’s Lynn, UK). Due to seasonal variations and operational limitations, barley plants were measured at different plant development stages, from the three leaves stage to end of tillering (Table 1). Chl *a* fluorescence is affected by chilling and freezing temperatures [44], thus screening was performed at temperature above 5 °C. For each line in the field or pot in the greenhouse, three plants were measured using the youngest fully emerged leaves. After 25 min of dark-adaptation using Hansatech leaf clips, fluorescence measurements were recorded for 2 s after illumination with a saturating light pulse of 3000 μmol photons m^−2^s^−1^ on the adaxial leaf surface. Fluorescence transients (Fig. 1) and JIP-test parameters [45] were extracted using PEA Plus Software (v1.10). The JIP-test parameters used for the study are summarized (Table 2). The maximum quantum efficiency of PSII (F_V_/F_M_) was used as the main stress indicator [46] as it has been demonstrated to be sensitive for Mn deficiency diagnosis [3, 6, 21]. However, Mn deficiency leads also to other marked changes in the transients [21]. Therefore, besides F_V_/F_M_, also the parameters V_I_, V_J_, V_K_ and Area were analysed as the main parameters for association genetic analysis (Table 2). A full Mn-containing design as control was not realized. Indeed in regards to the PSII and the quantum yield efficiency parameter measured (F_v_/F_m_), no diversity could be observed. Under those conditions, the quantum yield efficiency of PSII will reach its maximum with a value of F_v_/F_m_ around 0.82. Instead of such Mn-containing design, control pots (or lines) were sprayed with a solution of manganese sulphate (MnSO_4_) at 0.1 % of Mn containing two droplets of Tween® 20 detergent per litre as described by Schmidt *et al.* [6], in the field and greenhouse experiments. Plants were measured two days after spraying.

**Table 2:** Quantifying PSII performance parameters obtained from the JIP-test based on the Chl *a* fluorescence transients

Extracted and technical fluorescence parameters		
F_0_	=	F_50μs_, fluorescence intensity at 50 μs
F_K_	=	F_400μs_ fluorescence intensity at the K step (at 400 μs)
F_J_	=	F_2ms_, fluorescence intensity at the J step (at 2 ms)
F_I_	=	F_30ms_, fluorescence intensity at the I step (at 30 ms)
F_M_	=	Maximum fluorescence intensity
F_V_	=	(F_M_ – F_0_), variable fluorescence from a dark adapted leaf
F_V_/F_M_	=	Maximum quantum efficiency of PSII photochemistry.
tF_M_	=	Time to reach F_M_, in ms
V_K_	=	(F_K_ – F_0_) / (F_M_ – F_0_), relative variable fluorescence at 400 μs
V_J_	=	(F_J_ – F_0_) / (F_M_ – F_0_), relative variable fluorescence at 2 ms
V_I_	=	(F_I_ – F_0_) / (F_M_ – F_0_), relative variable fluorescence at 30 ms
Area	=	Area under the curve between F_0_ and F_M_

#### Mn concentration in leaf tissue

In LJ13, only the controls samples were harvested and decontaminated following the method described by Schmidt *et al.* [6] in order to remove MnSO_4_ excess on the leaves surface. In GH13A, all the plants were sampled. The three youngest fully emerged leaves of each plant in the pots were harvested and bulked. For the digestion of samples, 15-30 mg of freeze-dried leaves were mixed with 750 μL 70 % HNO_3_ (SCP science, Quebec, Canada) and 375 μL 15 % H_2_O_2_ (Sigma-Aldrich) and hereafter digested in a pressurised microwave oven (Ultrawave, Milestone Inc., Sorisole, Italy) for 10 min with a starting pressure of 40 bar and a temperature of 240 °C. After digestion, samples were diluted to 14 mL with Milli-Q water (Milli-Q Plus, Bedford, Massachusetts, USA) before measurement on an ICP-OES (Model 5100, Agilent Technologies, California, USA) equipped with a Meinhard nebuliser and cyclonic spray chamber. For quantification, an external 10 point calibration standard P/N 4400-132565 and 104 P/N 4400-ICP-MSCS (CPI International, Amsterdam, the Netherlands) was used. NIST 1515 Apple leaf certified reference material (National Institute of Standards and Technology, Gaithersburg, Maryland, USA) was analysed together with the samples to evaluate the accuracy and precision of the analysis. Data were accepted when the limit of quantification (LOQ) was exceeded and the accuracy was within ±10 % of the certified level and the coefficient of variation (CV%) among replicates was below 5 %. Four technical replicates were made for all samples and seven for the reference material. The contents of 13 elements were determined and expressed in μg.g^−1^ of leaf dry weight.

#### Phenotypic analysis

All phenotypic analyses were carried out using R version 3.1.0 [47]. To estimate the trait value across environments and replicates, estimates of the variance components were calculated using the general linear mixed model. Best linear unbiased predictors (BLUP) for each variety were calculated from a two-dimensional spatial mixed-model with measurement error using model from ASReml-R package [48, 49]. BLUPs are reported as being suitable for plant breeding trials [50, 51], moreover two-dimensional spatial analysis allows taking into account variation in Mn efficiency amongst field and greenhouse experiments. BLUPs were calculated within environments using the formula:

$$ {y}_{ij}=z+{g}_{w(i)}+{r}_{w(j)}+{v}_{w(ij)}+{e}_{w(ij)} $$

where _*w()*_ indicates within environment, *y*_*ij*_ is the response of the *i*-th variety for the *j*-th replicated-measurement, *z* is the intercept, *g*_*w*(*i*)_ is the *i*-th variety effect, *r*_*w*(*j*)_ is the *j*-th replicated measurement effect, *v*_*w*(*ij*)_ is the measurement error of the *ij*-th variety-replicated measurements. Finally, *e*_*w*(*ij*)_ is the residual error which fits separable first order autoregressive two-dimensional spatial process to the variance structure of the line (or pot) errors. The intercept is taken as fixed effect whereas other terms are random effects.

Genetic determination of the trait (H^2^) was calculated from the model according to the formula:

$$ {H}^2=\frac{{\widehat{\sigma}}_g^2}{{\widehat{\sigma}}_g^2+{\widehat{\sigma}}_r^2+{\widehat{\sigma}}_{\upupsilon}^2+{\widehat{\sigma}}_e^2} $$

where ${\widehat{\sigma}}_g^2$ is the genetic variance, ${\widehat{\sigma}}_r^2$ is the variance of replicated measurements, ${\widehat{\sigma}}_{\upupsilon}^2$ is the variance of measurement error and ${\widehat{\sigma}}_e^2$ is the residual variance.

### Genetic analyses

#### Collection genotyping

The whole population of winter barley varieties was genotyped with SNP markers by TraitGenetics GmbH (Gatersleben, Germany). Samples were genotyped using the Illumina® iSelect 9 k barley Infinium chip [52]. Allele calling was performed at TraitGenetics using the company’s own cluster file based on a diversity panel.

Intra-chromosomal positions used for the GWA were based on the physical map of barley [42] in order to locate the associated SNP on the genome. After pre-processing with minor allele frequencies (MAF) < 0.01 and with a call rate > 0.75, a final set of 5,706 SNPs was retained in the study, including 4,761 SNPs with known positions on the reference map [42].

#### Population structure calculation

The software package STRUCTURE [53] was used to infer the population structure. This approach uses multi-locus genotypic data to assign individuals to groups (k) without prior knowledge of population genetic relationship under Hardy-Weinberg equilibrium assumption. The calculation was done using the ParallelStructure R package [54]. STRUCTURE runs were distributed over 22 cores on a server to speed up the calculations. To determine the number of subpopulation (k), a first run was done, with a burn-in period of 25000 and 250000 Monte Carlo Markov Chain (MCMC) iterations, for k ranged from 1 to 10 with 15 independent replicates for each k. The admixture model with correlated allele frequencies was applied. The optimum number of subpopulation estimation was based on the ad hoc statistic (ΔK) [55] implemented in Structure Harvester website [56], based the ad hoc statistic (ΔK). The number of optimum subpopulation k was set to k = 4, therefore a new run was used to assign the genotypes to each subpopulation cluster with a setup with burn-in period of 100000, 500000 MCMC and 40 replicates for the estimate k. Results of independent runs for the same value of k were summarized using the CLUMPP software [57]. Estimation of membership coefficient was calculated with the Greedy permutation algorithm option with 100000 random input order repeats and plotted using Distruct software [58].

#### GWA model

The GAPIT R package was used for GWA [59]. The final GWA was performed using a modified mixed linear model taking. It takes into account multiple levels of relatedness and population matrix as cofactor [60, 61]. The pairwise relatedness matrix also called the genomic relationship or Kinship matrix was used to correct for relatedness in the GWA model and calculated using the Loiselle method [62]. Its dimension was (n x n), where n is the number of individuals. The developed model follows:

$$ \boldsymbol{Y}=X\boldsymbol{\beta} +Q\upupsilon +Z\boldsymbol{u}+\boldsymbol{e}\frac{1}{2} $$

where ***Y*** is the phenotypic response vector, *X* is the molecular marker matrix, ***β*** is the vector of fixed effect for the marker to be estimated, *Q* is the posterior probabilities matrix belonging to each population obtained for k = 4, υ is the vector of fixed effect for population structure, *Z* is the Kinship matrix, ***u*** is the vector of random effect for co-ancestry, and ***e*** is the vector of residuals. Only SNP markers with a P-value < 0.001 are presented in the results.

### Bioinformatics on candidate genes

SNPs associated with the Chl *a* fluorescence based parameters were blasted in the BARLEYMAP database [63]. The data-base refers to the barley physical map [42] and specify gene annotations upstream and downstream of the SNP in question. The blast window was extended to half the extent of linkage disequilibrium (LD) (Table 3) at both QTL region extremities, respectively and to each chromosome LD. In order to calculate LD extent, pairwise LD between loci was calculated in TASSEL version 5.0 [64] and the LD extent was implemented using the method described by Breseghello *et al.* [65].

**Table 3:** Intra-chromosomal extent of LD measured in the 248 winter barley collection

Chromosomes	Extent of LD (cM)	Number of SNPs
1H	6.5	490
2H	15.4	802
3H	14.7	736
4H	8.2	528
5H	14.3	870
6H	9.5	666
7H	18.5	669

## Results

### Selection of environments

Six different environments were used in the study to screen the 248 varieties of the winter barley collection. Mn deficiency induction cannot be easily controlled in fields and soil pots. Environmental effects such as temperature, soil humidity and redox states, all have major impacts on Mn plant availability and consequently on Mn deficiency development [66]. Consequently genotype-by-environment interactions were expected.

**Fig. 2:**
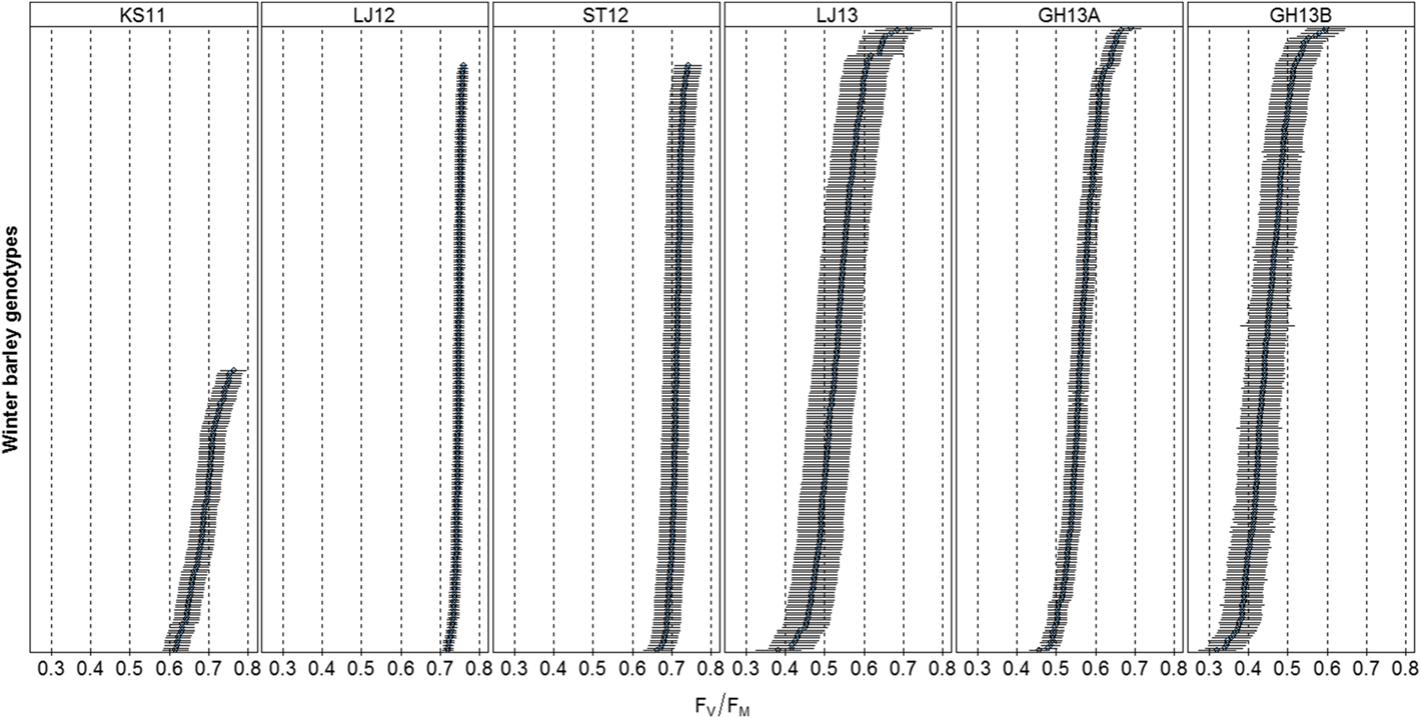
F_V_/F_M_ genotypic estimates presented by environments in the 248 winter barley population. Data represent the ranked BLUPs and their standard errors for each variety grown in Mn deficient soils

Therefore to study these interactions, genetic determination of the F_V_/F_M_ related trait, also called heritability (H^2^), and genetic correlation were calculated (Table 4). H^2^ characterizes the variance of the trait captured by the genotypic effects relative to the total phenotypic variance. The two field sites ST12 and LJ12 showed very low H^2^, at 0.06 for both whereas H^2^ was up to 0.31 for the greenhouse experiment GH13A. The low H^2^ estimated show that only a minor fraction of the total variance is due to genetic effects. In addition, when looking at F_V_/F_M_ genotypic estimates in the 6 environments (Fig. 2), the response range to low Mn availability ranged from 0.38 to 0.71 in LJ13 whereas LJ12 had F_V_/F_M_ values from 0.72 to 0.76 and ST12 from 0.66 to 0.74. Indeed LJ12 and ST12 were phenotype in spring whereas the other environments were screened in winter. The winter 2012/13 was too wet and mild to induce Mn deficiency, while it is induced mostly under dry and cold conditions. Consequently, because of low H^2^, narrow variability and inadequate phenotyping time, it was decided to discard ST12 and LJ12 from the GWA analysis.

**Table 4:** Genetic correlation between environments and genetic determination (H^2^) estimated by model for the F_V_/F_M_ trait

Environments	GH13A	GH13B	KS11	LJ12	LJ13	ST12	H^2^
GH13A	1						0.31
GH13B	0.61	1					0.26
KS11	−0.02	−0.11	1				0.18
LJ12	0.14	0.16	0.71	1			0.06
LJ13	0.27	0.28	0.16	0.25	1		0.23
ST12	0.11	0.14	0.24	0.84	0.2	1	0.06

Calculation of genetic correlations across environments (Table 4) provided a better understanding of how genetic control of Mn efficiency is related across the different environments. The weak genetic correlations confirmed that the Mn efficiency trait had differential genetic determination depending on environments also named genotype × environment interactions. These contrasts across environments can be explained by several factors including climate (solar irradiation, soil humidity and temperature) and physiological age of the plants relative to the time of Chl *a* measurements. Indeed, the severity of Mn deficiency is subject to major fluctuations over time and consequently it was also expected that the genetic variability among the varieties would be affected. Moreover, the ranking of variety’s F_v_/F_m_ performances fluctuated over the environments and no grouping of variety in regards to their origins or row-type was observed.

### Assessment of plant response to Mn deficiency

**Fig. 3:**
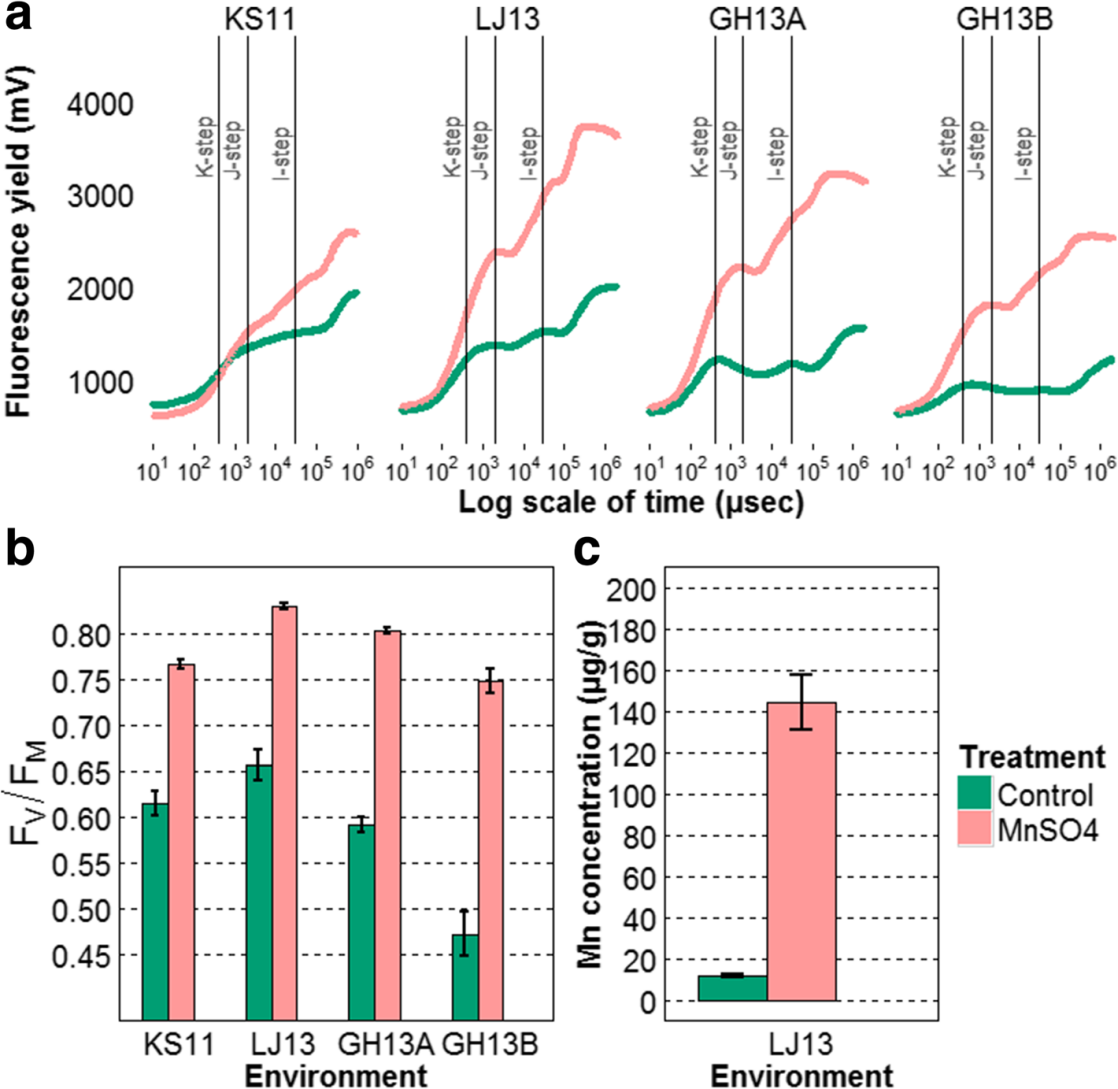
**a** Chl *a* fluorescence OJIP curves presented by treatments and environments (mean of replicates (24 ≤ n ≤ 45)). **b** F_V_/F_M_ values results calculated from Chl *a* fluorescence curves under different spraying treatments, and standard error over replicates are plotted with error bar. Finally, the plot (**c**) shows the Mn concentration in μg.g^−1^ of DW leaf tissue. In green, barley plants as controls did not receive any treatment application; in red, plants were sprayed with MnSO_4_ solution

To validate the induction of Mn deficiency, plants in soil pots and in fields were sprayed with soluble Mn and measured two days after spraying. In all four environments, a clear impact of MnSO_4_ foliar application was recorded on Chl *a* fluorescence curves and F_V_/F_M_ values (Fig. 3). The fluorescence yield F_V_/F_M_ of plants treated rose to a higher level (between 0.75 and 0.83) whereas the control plants remained at lower levels (between 0.47 and 0.66) (Fig. 3 b), confirming that the plants were indeed Mn deficient, but without any visual leaf symptoms (latent Mn deficiency). Therefore the ability to fully restore F_V_/F_M_ to its maximum after foliar application of Mn sulfate confirmed that Mn is the only abiotic stress parameters influencing the F_V_/F_M_ values under the conditions prevailing at the field sites and in the green house experiment. The J and I steps of the transients were flattened and less defined, and in severe cases a notable K-step was developed (Fig. 3 a). The most severe Mn deficient conditions were seen in the GH13B experiment in which Mn deficient plants showed flat J and I slopes. Furthermore, when Mn deficient plants in LJ13 were sprayed with MnSO_4_, the Mn concentration in leaf tissues increased significantly (Fig. 3 c). Therefore, spraying of Mn salts confirmed that Mn deficiency was induced and that plants can fully recover the maximum range of F_V_/F_M_. Moreover, the ICP-OES quantification of other nutrients indicated that their concentration in leaf tissues remained stable (Additional file 2). Consequently only Mn was responsible for the change of F_V_/F_M_.

Mn leaf concentration ranged from around 7 to 11.5 μgg^−1^ of DW and the H^2^ of Mn concentration in leaf tissue was 0.33. The Pearson correlation and its associated P-value showed the highest correlation between F_V_/F_M_ and Mn concentration at 0.59. The correlations were much lower with the other fluorescence parameters (Table 5).

**Table 5:** Correlations and the associated P-values of Mn concentration in leaf with Chl *a* fluorescence parameters

Fluorescence parameters	Correlation	P-value
F_V_/F_M_	0.59	<0.001
V_I_	0.32	<0.001
V_J_	0.03	0.659
Area	0.22	<0.001
V_K_	0.37	<0.001

Based on the differences in F_V_/F_M_, on the shape of the curves and on Mn leaf tissue concentration, it was evident that plants were exposed to Mn deficiency.

### Grouping from the population structure

**Fig. 4:**
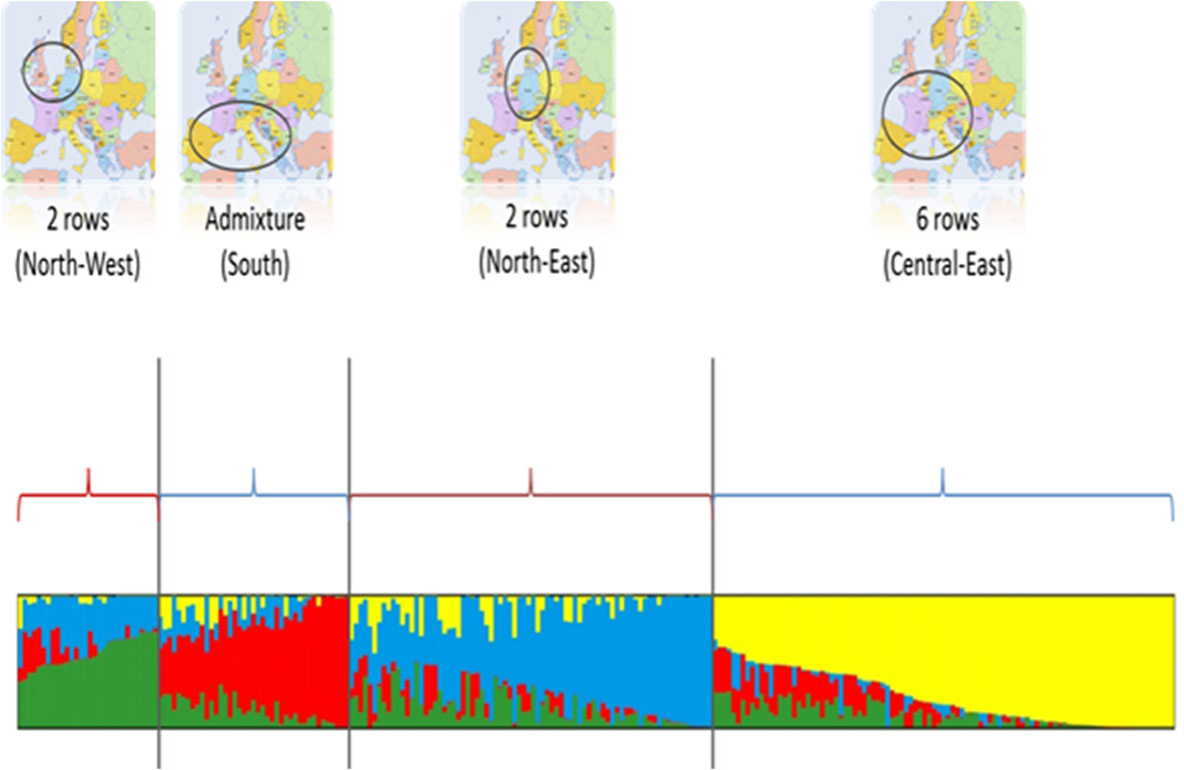
Allocation proportions of each variety to the four subpopulations. Each bar represents one variety, and each colour, one subpopulation

The structuration analysis allowed us to distinguish four groups (Fig. 4). Two groups of two-rows from mid-northern Europe were identified. One group of six-rows and one group with a mixture of six-rows and two-rows from a southern European origin were also detected. In blue, the group with two-rows contained most of the breeding lines of Scandinavian origin. It confirmed the significance of adding population structure into the GWA model.

### GWA results

**Fig. 5:**
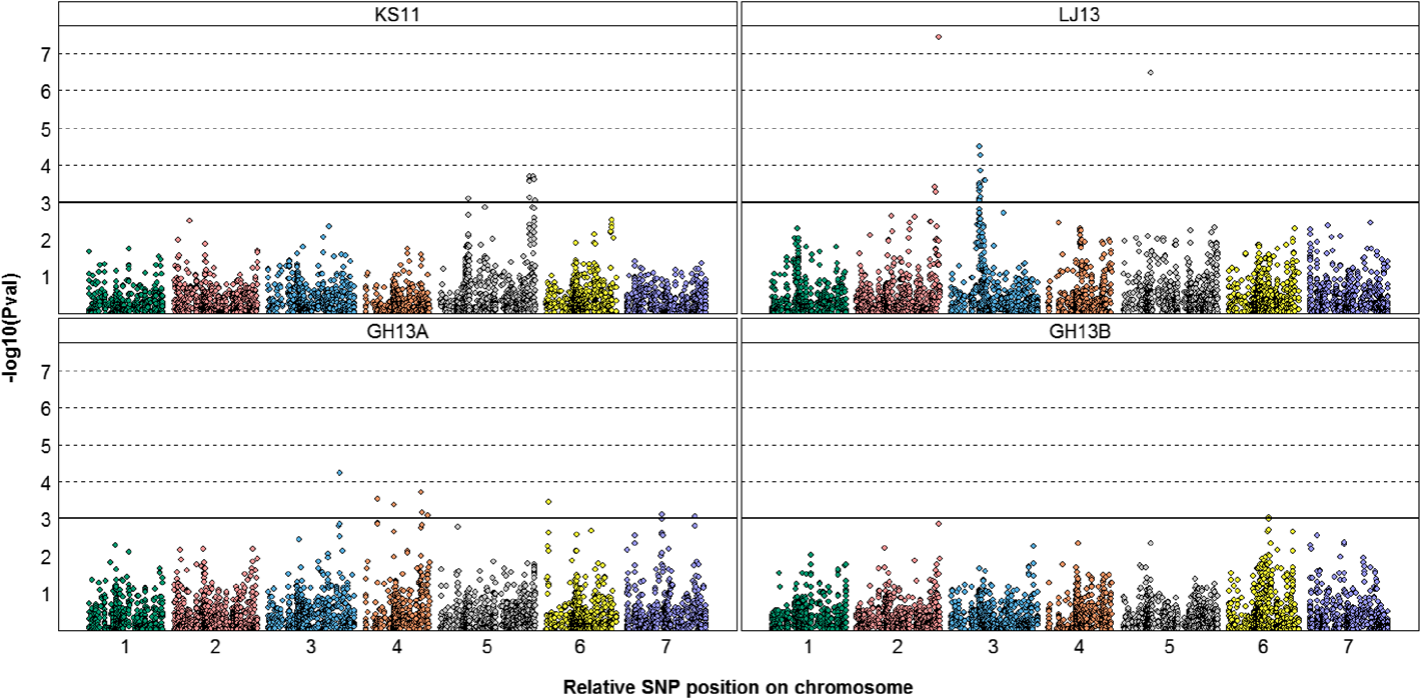
Manhattan plot of GWA on F_V_/F_M_ value for four environments. Each point represents one SNP over the seven barley chromosomes, with its chromosome position on x-axis and its –log_10_(P_Val_) associated on y-axis. The horizontal bar (-log_10_(P_Val_) = 3) represents a P-value of 0.001. The environments are field: KS11 and LJ13 and greenhouse GH13A and GH13B

Taken together for the four environments considered, 54 SNP markers were found to be significantly associated with the F_V_/F_M_ trait (P-value < 0.001) and were distributed over 6 chromosomes. Of these, 11 were unmapped SNPs with missing annotations. Marker-trait association displayed distinct peaks corresponding to QTLs (Fig. 5). Other associations were detected for all JIP parameters and are discussed below. Despite strong genotype × environment interactions, a meta-analysis including all environments (data not shown) resulted in the identification of the same variants. However it does not reflect the reality as most of the associations are environment-specific. Different SNPs were detected in each environment (Fig. 5) implying that marker-trait associations are severely affected by the environment. In contrast, only four SNPs were identified using the Mn leaf concentrations (Additional file 3). Three markers constitute one peak on chromosome 6H and one marker on chromosome 5H.

### Putative candidate genes

Recent advance in barley genome sequencing [42] allows candidate genes to be sought for in the flanking region of the SNP associations. Furthermore, the iSelect array was designed with SNP markers located in the genic region of known genes [67]. Therefore, the significant SNPs for each of the peaks were blasted against databases [63] to identify candidate genes in the surrounding region of associations. Putative candidate genes controlling Mn efficiency and their positions on the genome were identified in the corresponding blasted genic region. Based on prior knowledge about physiological mechanisms involving Mn, numerous genes coding for proteins have been reported and classified for F_V_/F_M_, Area, V_I_, V_J_ and V_K_ fluorescence parameters (Table 6 **,** Additional file 4). However, no putative genes have been reported for Mn leaf concentration data obtained by ICP-OES. It is suggested that all the candidate genes play a role in Mn dependent pathways and these are presented below.

**Table 6:** Summary of significant (−log_10_(P_Val_) ≥ 3) marker-trait associations identified by GWA based on the F_V_/F_M_ trait and the corresponding candidate genes from the Blast results

Environment	Marker name	Position		GWA statistics				BLAST results		
		Chr	cM	-log_10_(P)	MAF^a^	Effect	SE^b^	Gene	cM	Description
GH13A	12_30482	3H	128.6	4.2	0.26	0.014	0.003	**-**	**-**	**-**
	SCRI_RS_98443	4H	22.2	3.5	0.17	0.016	0.004	MLOC_75098.2	18.5	Cupin 1; Germin, Mn binding site
	12_30239		51.0	3.4	0.14	-0.015	0.004	MLOC_82113.1	49.7	PSII PsbN
								AK357955	54.3	Protein phosphatase 2C (PP2C) Mn/Mg aspartate binding site
	SCRI_RS_142924		51.0	3.4	0.14	-0.015	0.004	AK249774.1	54.3	PSII PsbP, oxygen evolving complex
	12_30987		100.6	3.7	0.43	0.012	0.003	MLOC_40094.1	99.1	PSI PsaA/PsaB
	SCRI_RS_131671		101.6	3.2	0.43	0.011	0.003			
	SCRI_RS_157125		111.3	3.1	0.13	-0.015	0.004	AK367749	111.3	Cupin 1; Germin, Mn binding site
	SCRI_RS_235762		111.3	3.1	0.13	-0.015	0.004			
	SCRI_RS_56	6H	4.9	3.4	0.30	0.011	0.003	-	-	-
	12_30149	7H	62.4	3.1	0.36	0.011	0.003	-	-	-
	SCRI_RS_184488		120.8	3.1	0.01	0.039	0.011	MLOC_3173.4	124.6	PP2C Mn/Mg aspartate binding site
								MLOC_77860.1	124.6	PSII PsbP, oxygen evolving complex
GH13B	SCRI_RS_144892	6H	72.2	3.1	0.30	-0.013	0.004	-	-	-
KS11	SCRI_RS_137249	5H	50.0	3.1	0.13	-0.019	0.005	AK368229	47.9	Chlorophyll *a/b* binding protein
								MLOC_18354.1	51.3	Chlorophyll *a/b* binding protein
	11_10254		159.5	3.7	0.13	-0.021	0.006	AK251925.1	159.5	PSII Psb28, class 1
	SCRI_RS_199298		159.5	3.1	0.13	0.019	0.006			
	SCRI_RS_235652		159.5	3.6	0.13	0.020	0.005			
	11_10870		159.5	3.6	0.12	0.022	0.006			
	SCRI_RS_237948		167.6	3.7	0.36	-0.015	0.004	-	-	-
	SCRI_RS_174123		167.7	3.6	0.31	-0.015	0.004			
	SCRI_RS_239569		169.4	3.0	0.50	-0.012	0.004			
LJ13	12_30823	2H	140.8	3.4	0.11	0.019	0.005	-	-	-
	SCRI_RS_73620		142.3	3.3	0.40	-0.015	0.004			
	SCRI_RS_231015		147.3	7.4	0.18	-0.027	0.005			
	12_30002	3H	51.4	3.5	0.33	-0.025	0.007	MLOC_38362.2	51.6	Chlorophyll *a/b* binding protein
	12_31372		51.4	3.5	0.32	-0.025	0.007			
	11_10365		51.6	4.5	0.32	0.029	0.007			
	SCRI_RS_173348		51.6	3.1	0.32	-0.023	0.007			
	11_20890		51.6	3.3	0.32	0.024	0.007			
	11_20102		51.6	3.5	0.32	0.025	0.007			
	SCRI_RS_137552		51.6	3.5	0.32	0.025	0.007			
	SCRI_RS_186504		51.6	3.5	0.32	0.025	0.007			
	11_10224		51.7	3.5	0.33	0.025	0.007			
	SCRI_RS_238114		51.8	3.4	0.33	-0.024	0.007	AK374059	52.6	PP2C Mn/Mg aspartate binding site
	11_10349		51.8	3.1	0.33	0.023	0.007			
	12_31011		52.1	3.1	0.35	0.019	0.006			
	12_31393		52.6	3.4	0.33	0.021	0.006			
	11_21511		52.6	4.3	0.35	0.025	0.006			
	11_20276		52.9	3.4	0.33	0.021	0.006			
	11_20325		53.1	3.1	0.32	-0.020	0.006			
	11_10225		53.3	3.8	0.33	-0.022	0.006			
	SCRI_RS_201987		59.6	3.6	0.28	0.016	0.004	**-**	**-**	**-**
	SCRI_RS_76971		61.7	3.6	0.28	0.016	0.004	AK369292	68.2	PSII PsbW, class 2
	11_21414	5H	49.9	6.5	0.21	0.023	0.004	AK368229	47.9	Chlorophyll *a/b* binding protein
								MLOC_18354.1	51.3	Chlorophyll *a/b* binding protein

^a^Minor Allele Frequency

^b^Standard error

Allelic effect sign is estimated with respect to the minor allele. SNPs are ordered by environment and genome position

#### Photosystem II subunits

Different Psb subunits from PSII were found at the exact position or close to the SNP associated with the trait by GWA. Of these, a highly significant association (-log_10_(P_val_) = 3.7) was found for F_V_/F_M_ in KS11 with SNP 11_10254 located on 5H chromosome at 159.5 cM. Gene AK251925.1 was found at the same position coding for Psb28 PSII subunit. Other marker-associations pointed out genes coding for PSII subunits; AK249774.1 (54.3 cM on chromosome 4H), MLOC_77860.1 (124.6 cM on chromosome 7H) coding for PsbP PSII subunit were also identified with a close co-localization of significant SNPs (Table 6). Besides these results, PsbW and PsbN (F_V_/F_M_ trait) and PsbQ (Area trait) PSII subunits coding genes were also found significant marker-associations, however they remained always more distant to the closest SNP.

#### Germin proteins

Germin and germin-like proteins (GLP) coding genes were identified in GH13A in two different positions on chromosome 4H. For the F_V_/F_M_ parameter (Table 6), SNP SCRI_RS_98443 was detected (-log_10_(P_val_) = 3.5) at 22.2 cM whereas the coding gene MLOC_75098.2 is located at 18.5 cM. The second association was identified by the SNPs SCRI_RS_157125 and SCRI_RS_235762 (-log_10_(P_val_) = 3.1) both at position 111.3 cM. The GLP coding gene AK367749 was detected at the same position.

#### Mn-SOD

The manganese superoxide dismutase (Mn-SOD) gene was identified in GH13A and LJ13 on chromosome 7H. Associations were found for V_I_ with SNP SCRI_RS_158266 located at 12.8 cM (-log_10_(P_val_) = 3.1) at the exact same position of the coding gene MLOC_69817.1 (Additional file 4).

#### Photosystem I subunits

Photosystem I (PSI) coding genes were found by GWA. Of these, PsaA/PsaB were found with the coding gene MLOC_40094.1 in GH13A for the F_V_/F_M_ trait on chromosome 4H at 99.1 cM. SNP 12_30987 was detected as the most significant association (-log_10_(P_val_) = 3.7) at the position 100.6 cM (Table 6). In addition to these QTLs, PsaH (MLOC_53469.2) and PsaE (MLOC_73050.2) were detected for V_I_ with SNPs 11_20908 (1H) and SCRI_RS_2824 respectively. They were 1 and 0 cM away from their respective genes. Finally, PsaN (AK368803) was identified with SNP 11_21057 (2H) at 0.1 cM from the marker position (Additional file 4).

#### Chlorophyll *a/b* binding protein

Genes coding for Chlorophyll *a/b* protein were identified for many associated SNPs on chromosomes 1H, 5H, 6H and 7H, in several copies and for all fluorescence parameters (Additional file 4). These proteins are also known as light-harvesting chlorophyll *a/b* binding (Lhc) proteins in higher plants and green algae.

#### Protein phosphatase 2C (PP2C) Mn/Mg aspartate binding

Many other SNPs were found to be significant and co-localized with PP2C genes. They were found in chromosomes 1H, 2H, 3H, 4H and 7H for all fluorescence parameters (Table 6 **,** Additional file 4). From the GWA results, multiple copies of these genes were identified.

## Discussion

### Quantification of Mn deficiency

Chl *a* fluorescence has previously been shown to be a powerful tool to quantify the severenes of Mn deficiency in plants [3, 6, 21]. However, little is known about differences in Mn uptake and Mn tissue concentrations in the plant tissue of varieties with contrasting Mn efficiency. Therefore, the quantification of Mn in leaves was used as an additional parameter.in the current study. Plants were clearly Mn deficient as indicated by the leaf tissue concentrations ranging from7-12 μg.^−1^ Mn g DW [21].

However, the correlation, 0.59, between Mn concentration and F_V_/F_M_ does not suggest a strong link between the two parameters. GWA did not reveal any strong association with tissue Mn as indicated by the absence of a correlation between leaf Mn concentrations and the functional pool of Mn in PSII using Fv/Fm values as a proxy.

### Environment-specific QTLs

Mn efficiency appears to be a complex quantitative trait controlled by many genes, each contributing with minor effects. Genetic determination of the trait ranged between 0.18 and 0.31 in the four environments analysed (Table 4), which means that only 18 % to 31 % of the phenotypic variance was due to the genetic variance. This supports the variability of plant responses to Mn deficiency partly being determined by genetic background. However, in contrast to traits governed by a few loci with large effects, the variation in quantitative traits is caused by the segregation of multiple QTL with individually small effects that are sensitive to the environment [68]. Indeed, significantly associated SNPs were found with relatively small effects (0.011 to 0.039) for F_V_/F_M_ (Table 6). Those markers are identified only within specific environments and the corresponding QTLs are consequently specifically related to a single environment. Collins et *al.* [13] introduced the concept of “constitutive” and “adaptive” QTLs controlling the response of crops to abiotic stress. A “constitutive” QTL is consistently detected across different environments whereas “adaptive” QTL is environment-specific. Stress sensitivity can therefore be due to the responsiveness of QTL regulation in specific environmental conditions or due to indirect causes contributing to the stress response. Staple or constitutive QTLs are naturally preferred in breeding.

In association mapping, detecting constitutive QTLs for abiotic stresses is challenging, since QTL effects are most likely small and controlled by one or several genes, and by epistasis and environments. Environment-specific QTL has been previously reported in cereals, in winter barley [69], in wheat [70, 71], and in rice [72]. In the case of the Mn efficiency trait, the four environments included, had varying characteristics due to various climates in the three different years, differences in soil conditions and/or different growth stages at the screening date (Table 1).

Moreover, poor genetic correlations between environments also support the environment-specific feature of the trait, with correlations in a range from -0.11 to 0.61 for the four environments analysed (Table 4). Nevertheless, with a genetic correlation of 0.61, the two greenhouse experiments imply that Mn efficiency in GH13A and GH13B was regulated by a set of shared genes under the controlled environmental conditions, with identical soil and comparable climates.

The results confirmed that environments have to be considered independently for the complex Mn efficiency trait. Consequently, different environmental conditions are needed to obtain a comprehensive picture of the trait and to identify the “adaptive” QTLs contributing to Mn efficiency.

### Identification of candidate genes

Combining GWA, database screening and prior knowledge about the role of Mn in plants allowed several candidate genes to be identified, coding for PSI and PSII proteins, chlorophyll *a/b* binding proteins, GLP, Mn-SOD, and PP2C. Two different classes of encoding genes could be distinguished, the first related to photosynthesis and the others involved in various Mn dependent pathways.

In photosynthesis, reactions occur in the thylakoid membranes carried out by multi-protein complexes, namely ATP synthase, PSI, PSII, cytochrome *b6f* and light harvesting complexes. Proteins from these complexes were identified: chlorophyll *a/b* binding proteins, PsaA/PsaB, PsaE, PsaH and PsaN from PSI; Psb28, PsbN, PsbP, PsbQ and PsbW from PSII; and finally Lhc proteins in multiple copies at several genome positions.

### Role of Mn in metabolic pathways

The light-harvesting chlorophyll *a/b* binding proteins belong to the Lhc family consisting of more than 20 different proteins and are connected with PSI and PSII [73]. Their primary function is related to the absorption of light through chlorophyll excitation and the transfer of the absorbed energy to photochemical reaction centres. Although Lhc are coded by well characterized and abundant CAB (chlorophyll a/b binding) genes [74, 75], the link to Mn metabolism is not trivial. Nevertheless, previous studies showed a reduction in Chl *a* concentration in Mn deficient plants and changes in the phosphorylated forms of LhcII have been observed [21].

Several protein subunits from PSI were also detected. Similarly to the chlorophyll *a/b* binding proteins there is no obvious and immediate links to Mn utilization. The subunits PsaA/PsaB are involved in electron transport and form the core centre of PSI [76]. PsaE is one of the three stromal subunits of PSI whereas PsaH and PsaN are specific plants subunits [77, 78]. Although Mn is not a PSI cofactor, a decrease in the PSI activity in Mn-limited conditions has been reported previously. The composition of the PSI complex is altered and a decrease in PsaA subunit is pronounced [79].

PSII consists of at least 20 protein subunits and catalyses a light-driven process, splitting water into protons and molecular oxygen. [75, 80]. The Psb28 protein is associated with the cytoplasmic side of the thylakoid membrane and known to interact with the core protein CP43 in the PSII monomer [81]. Even though no direct link with Mn has been demonstrated, recent studies have partly established its function. A Psb28 deletion mutant generated in *Synechocystis* showed slower growth rates and lower chlorophyll levels. [81, 82]. Recent studies suggest a role of Psb28 in the assembly and repair of PSII under heat stress [83, 84].

In barley, PsbP and PsbQ subunits are extrinsic proteins involved in the stabilization of the tetranuclear Mn cluster of OEC. [85, 86]. The crucial role of PsbP in Mn binding to PSII has already been established [87]. It has been described as a Mn storage protein delivering Mn^2+^ during PSII assembly. Its absence slows down the process of photoactivation. Whereas PsbP was found mandatory for OEC activity and showed a strong Mn dependence, PsbQ did not present a manganese-dependent activity [88]. However, recent studies established that PsbQ stabilizes PsbP binding and therefore contributes to the activity of the OEC in PSII complexes [89]. Finally, a study on the tobacco PsbQ mutant revealed the effect of PsbQ loss on Chl *a* fluorescence under low light conditions. For the PsbQ mutant, F_V_/F_M_ dropped from 0.73 to 0.26 under low light conditions [90]. Hence, measuring Mn efficiency based on Chl *a* fluorescence appears to be linked to PsbQ activity. PsbN, a low molecular subunit encoded in the chloroplast, has exclusively been found in etioplasts, but its PSII function remains elusive [91]. Nevertheless, a recent study has demonstrated by reverse genetics in tobacco, that PsbN is involved in the biogenesis of PSI and PSII [92]. In addition, it has been demonstrated that PsbN is required for the repair and assembly of PSII [93].

Recent work with PsbW knock-out and antisense Arabidopsis mutants indicated an important role played by PsbW for the connection and stability of PSII-LHCII complexes [94]. Lower F_V_/F_M_ values have been reported for mutants lacking PsbW, but despite the role of PsbW for the PSII complex, direct effects of Mn on PsbW remain unclear.

The identification of several candidate genes coding for PSII subunit proteins (PsbN, PsbP, PsbQ and PsbW) demonstrate that Mn is involved in the stability, in the assembly and repair of the PSII and in the loading of Mn into the photosystem machinery. Therefore, it suggests that some varieties are able to load Mn, assembly PSII and repair PSII better than other under Mn deficient condition.

Apart from photosystem related proteins, two other types of proteins were found to be involved in Mn efficiency: PP2C and GLP. PP2C is a well-known serine/threonine specific protein phosphatase. With 76 candidate genes identified in Arabidopsis [95], the PP2C family is considered the largest protein phosphatase family in plants. They are identified on several barley chromosomes (1H, 2H, 3H, 4H and 7H). These phosphatases are involved in the regulation of several signalling pathways and stress responses in plants [96]. In the current context, an interesting feature of PP2C is the relatively high concentration of Mn or Mg required to maintain activity. In moss *Physcomitrella patens*, the activation of PP2C occurs at 0.1 mM of Mn whereas more than 5 mM of Mg was needed to activate the protein [97]. It therefore suggests that Mn has a special feature in activating PP2C, which appears to be a factor influencing Mn efficiency.

The remaining genes identified by GWA are coding for GLP and Mn-SOD. GLP are often described in crops as having two main enzymatic functions, OxO and SOD, in response to biotic and abiotic stress [98, 99]. The two enzymes, Mn-SOD and OxO, generate hydrogen peroxide (H_2_O_2_), a signalling molecule in plant defence. OxO enzymes are localized in the apoplast and Mn-SOD in mitochondria; they work in antioxidant defence by enzymatic mechanisms scavenging reactive oxygen species (ROS). A first study demonstrated the involvement of Mn as a cofactor in barley OxO activity [26]. Hence, it has been confirmed by spectroscopy and crystallography that Mn is a required cofactor for OxO catalysis [100, 101]; moreover it revealed the Mn-SOD activity of GLP. GLP coding genes in barley are mapped on chromosomes 2H, 3H, 4H, 5H, 7H and classifies to five main subfamilies annotated *Hv*GER-I to *Hv*GER-V [102]. Finally, gene family studies on barley [103, 104] confirmed the OxO and SOD activity of GLP, their function in defence resistance, the duplication of GLP genes, their developmental stage and their tissue-specific expression. The excellent characterization of the barley GLP genes and activity may contribute to a better understanding of the role of Mn.

## Conclusions

Mn deficiency was the sole cause for the decrease in quantum yield efficiency (F_V_/F_M_). The combination of Chl *a* fluorescence analysis and GWA approaches used in the current study has unravelled a series of main components contributing to the polygenic trait for Mn efficiency and paves the way for a better genetic characterization of the trait. Several putative QTLs anchored in various chromosomes pointed out genes coding for proteins which have a clear Mn dependency. First of all, multiple PSI and PSII candidate genes were identified of which PsbP is known to be directly involved in a Mn dependent regulation of PSII activity, whereas other photosystem subunits PsbQ, PsbN and Psb28 are important for maintaining the catalytic properties of the Mn cofactor in OEC r. Furthermore, two other types of genes coding for Mn dependent proteins were identified: GLP and PP2C genes. Both proteins are involved in stress signalling pathways. Even though the candidate genes needs to be confirmed by molecular approaches such as fine mapping, gene expression or mutant studies, the current study identifies a series of important candidate genes, suitable for future investigations into the genetic components controlling differential Mn efficiency in plants.

## Additional files

Additional file 1: List of the 248 winter barley varieties. (PDF 24 kb)
Additional file 2: Figure of elemental profiles of the controls plants in LJ13 in μg.g^−1^ of leaf dry weight and their standard error bars. (PDF 20 kb)
Additional file 3: Table of GWA results for Mn concentration. (PDF 13 kb)
Additional file 4: Table of GWA results for JIP parameters. (PDF 49 kb)

## Abbreviations

BLUP: Best linear unbiased predictors
Chl *a*: Chlorophyll *a*
CV%: Coefficient of variation
GLP: Germin-like protein
GWA: Genome-wide association study
H^2^: Genetic determination of the trait
ICP-OES: Inductively coupled plasma - optical emission spectrometry
LD: Linkage disequilibrium
Lhc: Light-harvesting chlorophyll a/b binding
LOQ: Limit of quantification
MAF: Minor allele frequencies
Mn: Manganese
Mn-SOD: Mn superoxide dismutase
OEC: Oxygen-evolving complex
OxO: Oxalate-oxidase
PAL: Phenylalanine ammonia-lyase
PP2C: Protein phosphatase 2C
PSI: Photosystem I
PSII: Photosystem II
ROS: Reactive oxygen species
SNP: Single nucleotide polymorphism
