# Erratum to: Identification of manganese efficiency candidate genes in winter barley (*Hordeum vulgare*) using genome wide association mapping

**DOI:** 10.1186/s12864-016-3165-5

**Published:** 2016-10-18

**Authors:** Florian Leplat, Pai Rosager Pedas, Søren Kjærsgaard Rasmussen, Søren Husted

**Affiliations:** Department of Plant and Environmental Sciences, University of Copenhagen, Thorvaldsensvej 40, 1871 Frederiksberg, Copenhagen Denmark

## Erratum

*n.b. The errors and associated corrections described in this document concerning the original manuscript were accountable to the production department handling this manuscript, and thus are no fault of the authors of this paper. Additionally, the online manuscript has now been updated with these corrections accordingly.*

In the original publication of this article [1], there was a mistake in the formula within the ‘GWA model’ section.

The formula below is incorrect:

$$ \boldsymbol{Y}=X\boldsymbol{\beta} +Q\upupsilon +Z\boldsymbol{u}+\boldsymbol{e}\frac{1}{2} $$

This should be:

$$ \boldsymbol{Y}=X\boldsymbol{\beta} +Q\upupsilon +Z\boldsymbol{u}+\boldsymbol{e} $$
