# Supplementary material for: Identification of manganese efficiency candidate genes in winter barley (Hordeum vulgare) using genome wide association mapping
Source: BMC Genomics. 2016 Oct 4;17:775. doi: 10.1186/s12864-016-3129-9 (PMC5050567; doi:10.1186/s12864-016-3129-9)
Supplement: Additional file 1: — List of the 248 winter barley varieties. (PDF 24 kb) [file 12864_2016_3129_MOESM1_ESM.pdf]

Additional file 1

List of the 248 winter barley varieties

Varieties	Collection origin	Row-type	Released decade	Country of origin	Breeder's origin
**Abondant**		6	2000	IT	Cambier Semences
**Aci**	Exbardiv	2	1990	IT	Istituto sperimentale per la cerealicoltura
**Ager**		6	1963	FR	INRA
**Aiace**	Exbardiv	2	2000	IT	Cecop
**Airone**	Exbardiv	2	2000	IT	Istituto sperimentale per la cerealicoltura
**Ajour**		2	2000	SK	Sejet
**Akropolis**		6	2000	AT	Saatzucht Firlbeck
**Alce**	Exbardiv	2	2000	IT	Istituto sperimentale per la cerealicoltura
**Aldebaran**	Exbardiv	6	2000	IT	D.J. Van der Have
**Alfeo**	Exbardiv	2	2000	IT	Istituto sperimentale per la cerealicoltura
**Alpha**		2	1970	FR	INRA
**Amarena**		6	2000	FR	Saaten Union
**Amillis**	Exbardiv	2	2000	FR	Limagrain Europe
**Angela**		6	1990	FR	INRA
**Anisette**		2	2000	DK	Sejet
**Antonella**		6	2010	DE	Nordsaat Saatzucht
**Antonia**		2	1990	FR	Secobra
**Apropos**		2	2000	DK	Sejet
**Aquarelle**	Exbardiv	2	2000	DE	Lochow-Petkus
**Arcanda**		2	2000	AT	Saatzucht Donau
**Arco**		2	1990	FR	Secobra
**Arda**	Exbardiv	2	1980	IT	Istituto sperimentale per la cerealicoltura
**Arma**	Exbardiv	6	1970	FR	Florimond Desprez
**Arturio**		6	2000	FR	Secobra
**Asso**	Exbardiv	2	1990	IT	Società Produttori Sementi Bologna
**Athene**	Exbardiv	6	1970	DE	Eckendorf
**Augusta**		2	2010	DE	Sejet
**Aydanhanim**	Exbardiv	2	2000	TR	Central Research Institute for Field Crops
**Azurel**		6	2000	FR	Secobra
**Balaki**	Exbardiv	6	1990	FR	Secobra
**Balda**	Exbardiv	6	2000	IT	APSOV
**Balkan**		6	1990	FR	Nickerson
**Baraka**	Exbardiv	2	1980	FR	Serasem
**Barberousse**		6	1970	FR	Ringot
**California**		2	2010	UK	Limagrain UK Ltd
**Camera**		2	2000	AT	Nickerson UK Ltd
**Camion**		2	2000	UK	KWS UK
**Campanile**		2	2000	UK	Nickerson UK Ltd
**Canoro**	Exbardiv	6	1990	IT	Secobra
**Cantare**		2	2000	DE	Limagrain GmbH
**Carat**	Exbardiv	2	2000	UK	Nickerson UK Ltd
**Caravan**		2	2000	FR	Limagrain Europe
**Carola**	Exbardiv	6	1990	DE	Nordsaat Saatzucht
**Casino**		6	2010	FR	Momont Hennette et Fils
**Cassiopee**		2	2010	FR	Secobra

**Cervoise**		6	2000	FR	Momont Hennette et Fils
**Chalup**		2	2010	DK	Sejet
**Champie**		6	2000	FR	Florimond Desprez
**Clara**	Exbardiv	2	2000	DE	Nordsaat Saatzucht
**Clarine**	Exbardiv	2	2000	FR	Secobra
**Colibri**		6	2000	FR	Momont Hennette et Fils
**Cordula**		2	2000	AT	Saatzucht Donau
**Crimont**		6	1980	IT	
**Criter**		6	1980	FR	Nickerson
**Dahlia**		6	1980	FR	
**Daniela**		2	2010	DK	Sejet
**Dasio**	Exbardiv	2	1990	IT	Istituto sperimentale per la cerealicoltura
**Dea**	Exbardiv	6	1950	DE	Engelen
**Declic**		6	2010	FR	Ets Jean Lemaire-Deffontaines
**Diadem**	Exbardiv	2	2000	FR	Serasem
**Djebel**		6	2000	FR	Serasem
**Dolmen**	Exbardiv	2	2000	FR	Momont Hennette et Fils
**Duchess**	Exbardiv	2	1990	FR	Ets Jean Lemaire-Deffontaines
**Duet**	Exbardiv	2	1990	UK	Nickerson UK Ltd
**Dura**	Exbardiv	6	1960	DE	Streng
**Esterel**	Exbardiv	6	1990	FR	Secobra
**Etincel**		6	2010	FR	Secobra
**Etrusco**		6	1980	IT	Federconsorz / SIS
**Express**	Exbardiv	6	1990	FR	Serasem
**Famosa**		2	2010	DE	Nordic Seed / Breun
**Fanfare**	Exbardiv	2	1990	UK	ICI-Seeds
**Federal**	Exbardiv	6	1990	FR	Secobra
**Fighter**	Exbardiv	2	1990	UK	Limagrain UK Ltd
**Finesse**	Exbardiv	2	1990	UK	ICI-Seeds
**Finlissa**		2	2000	DK	Nordic Seed
**Fjord**		2	1990	FR	Secobra
**Flagon**		2	2000	UK	Syngenta UK
**Florentine**		2	2000	UK	KWS UK
**FM-9737**		6	2000	DE	Syngenta DE
**Franka**	Exbardiv	6	1980	DE	Streng / IG Pflanzenzucht
**Fridericus**	Exbardiv	6	2000	DE	KWS Lochow
**Frost**	Exbardiv	6	1990	SWE	Weibull
**Gaiano**		6	1990	IT	Società Italiana Sementi
**Gerbel**	Exbardiv	6	1980	FR	Florimond Desprez
**Gigga**		6	2010	FR	Momont Hennette et Fils
**Gleam**	Exbardiv	2	1990	UK	CC Benoist / New Farm Crops
**Glenan**	Exbardiv	6	1980	FR	Nickerson
**Gloria**		2	2000	AT	Saatzucht Streng's Erben
**Gospel**		2	2010	DK	Sejet
**Gotic**		6	1990	FR	Florimond Desprez
**Grete**	Exbardiv	6	1990	DE	Eckendorf
**Gudrun**		2	1990	AT	Landwirtschaftliche Fachschule Edelhof
**Halcyon**	Exbardiv	2	1970	UK	PBI
**Hannelore**		2	2000	AT	Saatzucht Donau
**Hasso**	Exbardiv	6	1980	DE	Lochow-Petkus
**Hatif-de-**	Exbardiv	6	1940	FR	Grignon
**Heligan**	Exbardiv	2	2000	UK	CPB / KWS UK Limited
**Henriette**		6	2010	DE	Nordsaat Saatzucht
**Herfordia**	Exbardiv	6	1960	DE	Dippe
**Highlight**		6	2000	DE	DSV
**Holmes**		6	2010	DK	Nordic Seed
**Hoppel**	Exbardiv	6	1970	FR	Blondeau
**Isa**	Exbardiv	6	1980	BE	
**Isacco**		6	1990	IT	INRA
**Isocel**		6	2010	FR	Secobra
**Jade**		2	2010	DE	KWS UK

**Jaidor**	Exbardiv	6	1980	FR	INRA
**Jewel**	Exbardiv	2	1990	UK	Nickerson UK Ltd
**Kaskade**	Exbardiv	2	1980	DE	BPZ-Saatenring
**Katja**		2	1970	AT	Saatzucht Donau
**Kelibia**	Exbardiv	2	1990	FR	Limagrain Europe
**Kestrel**	Exbardiv	2	1990	UK	Breun
**Ketos**	Exbardiv	6	2000	FR	Limagrain Europe
**KWS-Ariane**		2	2010	DE	KWS Lochow
**KWS-Cassia**		2	2010	UK	KWS UK
**KWS-Glacier-**		2	2010	UK	KWS UK
**KWS-Joy**		2	2010	DE	KWS Lochow
**KWS-Liga**		2	2010	DE	KWS Lochow
**KWS-Meridian**		6	2010	DE	KWS Lochow
**KWS-Scala**		2	2010	DE	KWS Lochow
**KWS-Tenor**		6	2010	DE	KWS Lochow
**Labea**	Exbardiv	2	1990	DE	Breun
**Laverda**	Exbardiv	6	2000	DE	Nordsaat Saatzucht
**Leibniz**		6	2000	DE	KWS Lochow
**Leonie**	Exbardiv	2	2000	UK	Nordsaat Saatzucht
**Letizia**		6	2000	IT	Istituto sperimentale per la cerealicoltura
**Lombard**		2	1990	IT	Florimond Desprez
**Lomerit**		6	2000	DE	KWS Lochow
**Lonni**	Exbardiv	6	2000	DK	Pajbjergfonden
**Lorena**	Exbardiv	6	1990	DE	Saaten-Ring
**Ludmilla**	Exbardiv	6	2000	DE	Saatzucht Firlbeck
**Lutece**	Exbardiv	6	2000	IT	Serasem
**Magie**	Exbardiv	2	1990	FR	Serasem / Ackermann
**Majestic**		6	1990	FR	Unisigma
**Malice**		2	2000	FR	Unisigma
**Malicorne**		2	2000	FR	Unisigma
**Malta**	Exbardiv	2	1970	DE	Ackermann Saatzucht
**Malwinta**	Exbardiv	2	2000	DK	Pajbjergfonden
**Manitou**	Exbardiv	6	1990	FR	Secobra
**Manolia**	Exbardiv	2	2000	FR	Secobra
**Marado**	Exbardiv	6	2000	FR	Serasem
**Marinka**	Exbardiv	2	1990	NLD	Cebeco
**Maris-Otter**	Exbardiv	2	1960	UK	Robin Appel Ltd / PBI
**Maris-Trojan**	Exbardiv	2	1970	UK	PBI
**Matros**		2	2010	DK	Sejet
**Mattina**	Exbardiv	6		FR	INRA
**Melanie**	Exbardiv	2	1990	DE	Breun
**Menhir**		2	2000	FR	RAGT / Engelen
**Mercedes**		6	2000	DE	Secobra
**Metaxa**		2	2000	DE	Ackermann Saatzucht
**MH-Firenzza**		2	2010	DE	KWS Lochow
**Micuccio**		6	1970	IT	Pepe-Gravina
**Mirco**	Exbardiv	6	1980	IT	Coopsementi srl / Sassano
**Mirra**	Exbardiv	6	1970	DE	Eckendorf
**Montana**	Exbardiv	2	1990	NLD	Probstdorfer Saatzucht
**Murcie**	Exbardiv	2	2000	FR	Florimond Desprez
**Muscat**	Exbardiv	6	1990	UK	CPB / KWS UK Limited
**Naomie**	Exbardiv	6	2000	DE	Ackermann Saatzucht
**Nerz**		6	2000	DE	KWS Lochow
**Nikel**		6	1990	DE	Eckendorf / Serasem
**Nure**	Exbardiv	2	2000	IT	Istituto sperimentale per la cerealicoltura
**Onice**	Exbardiv	6	1980	IT	Società Italiana Sementi
**Opal**	Exbardiv	2	2000	DE	Nickerson UK Ltd
**Orbise**		2	2000	FR	RAGT
**Orchidea**		2	2000	IT	GEA Sementi
**Ordinale**		2	2010	FR	RAGT
**Orjoie**		2	2010	FR	RAGT

**Panda**	Exbardiv	2	1980	FR	Desprez
**Passerel**		6	2010	FR	Secobra
**Passport**	Exbardiv	6	2000	FR	Lemaire Deffontaines
**Pastoral**	Exbardiv	2	1990	FR	Secobra
**Patricia**	Exbardiv	6	1990	AT	Probstdorfer Saatzucht
**Pearl**	Exbardiv	2	2000	UK	Nickerson UK Ltd
**Pelican**		6	2000	FR	Saaten Union
**Perga**		6	1960	DE	Heine-Peragis
**Pilastro**	Exbardiv	6	1990	IT	Società Italiana Sementi
**Pirate**	Exbardiv	6	1980	FR	Ringot
**Plaisant**	Exbardiv	6	1980	FR	GAE
**Platine**	Exbardiv	2	2000	FR	Serasem
**Pompadour**		2	2010	FR	Cambridge Plant Breeders / RAGT
**Ponente**	Exbardiv	6		IT	Cecop
**Precosa**		2	2010	AT	Saatzucht Dr. Hans Hege
**Princess**	Exbardiv	6	1990	FR	Lemaire Deffontaines
**Puffin**	Exbardiv	2	1990	UK	ICI-Seeds
**Red**		2	1990	IT	Coopsementi srl
**Reflexion**		6	2010	FR	Lemaire Deffontaines
**Retriever**		2	2000	UK	KWS UK
**Robur**	Exbardiv	6	1970	FR	INRA
**Roseval**		6	2010	BE	Jorion
**Saffron**		2	2000	UK	KWS UK
**Saigon**	Exbardiv	2	2000	UK	Nickerson UK Ltd
**Salamandre**		2	2010	FR	Secobra
**Salling**		2	2000	UK	Limagrain UK Ltd
**Samson**		6	1990	IT	Cambier Semences
**Selvaggio**		6	1980	IT	
**Senta**	Exbardiv	6	1960	DE	Engelen
**Sequel**		6	2000	UK	Syngenta UK
**Sereno**		6	2010	IT	Societa Agricola Tara di Andrea Guerresi
**Siberia**		6	2000	FR	Secobra
**Sinatra**		2	2010	UK	Sejet
**SJ-NUE16**	Sejet	2	2010	DK	Sejet
**SJ-NUE17**	Sejet	2	2010	DK	Sejet
**SJ-NUE18**	Sejet	2	2010	DK	Sejet
**SJ-NUE19**	Sejet	2	2010	DK	Sejet
**SJ-NUE20**	Sejet	2	2010	DK	Sejet
**SJ-NUE21**	Sejet	2	2010	DK	Sejet
**SJ-NUE22**	Sejet	2	2010	DK	Sejet
**SJ-NUE23**	Sejet	2	2010	DK	Sejet
**SJ-NUE24**	Sejet	2	2010	DK	Sejet
**SJ-NUE25**	Sejet	2	2010	DK	Sejet
**SJ-NUE26**	Sejet	2	2010	DK	Sejet
**SJ-NUE27**	Sejet	2	2010	DK	Sejet
**SJ-NUE28**	Sejet	2	2010	DK	Sejet
**SJ-NUE29**	Sejet	2	2010	DK	Sejet
**SJ-NUE30**	Sejet	2	2010	DK	Sejet
**SJ-NUE31**	Sejet	2	2010	DK	Sejet
**SJ-NUE32**	Sejet	2	2010	DK	Sejet
**SJ-NUE33**	Sejet	2	2010	DK	Sejet
**Skamling**		2	2010	DK	Ackermann Saatzucht / Nordic Seed
**Sonja**	Exbardiv	2	1970	DE	Engelen / Secobra
**Sonora**	Exbardiv	6	1990	FR	Nickerson
**Souleyka**		6	2010	FR	Lemaire Deffontaines / Nordsaat Saatzucht
**Sprite**	Exbardiv	2	1990	UK	PBI
**Talisman**		2	2010	DK	Sejet
**Tamaris**		6	1990	FR	Secobra
**Tapir**	Exbardiv	6	1980	NLD	Semundo BV / HAEG
**Target**	Exbardiv	2	1990	UK	New Farm Crops
**Theresa**		6	1990	FR	Secobra

**Tiffany**	Exbardiv	2	2000	DE	Breun
**Tipper**		2	1980	UK	Nickerson UK Ltd
**Torrent**	Exbardiv	2	1980	UK	Nickerson / RPB
**Touareg**		6	2010	FR	Lemaire Deffontaines
**Trasimeno**	Exbardiv	2		YUG	Poljoprivredni Institut
**Trebbia**	Exbardiv	6	1990	IT	Istituto sperimentale per la cerealicoltura
**Tria**	Exbardiv	2	1960	DE	Ackermann Saatzucht
**Ultra**		2	1980	IT	Firlbeck
**Vanessa**	Exbardiv	2	2000	DE	Breun
**Verticale**	Exbardiv	2	2000	FR	Serasem
**Vertige**		2	1990	FR	Serasem
**Vetulio**		6	1980	IT	
**Vogelsanger-**	Exbardiv	6	1960	DE	Hauptsaaten
**Winsome**		2	2010	UK	Syngenta UK
**Wintmalt**		2	2000	UK	KWS Lochow
**Yatzy**		2	2010	DK	Sejet
**Yokohama**		6	2010	DE	DSV
**Zacinto**	Exbardiv	2	2000	IT	Istituto sperimentale per la cerealicoltura
**Zoe**	Exbardiv	6	2000	DE	
