# Supplementary material for: Identification of manganese efficiency candidate genes in winter barley (Hordeum vulgare) using genome wide association mapping
Source: BMC Genomics. 2016 Oct 4;17:775. doi: 10.1186/s12864-016-3129-9 (PMC5050567; doi:10.1186/s12864-016-3129-9)
Supplement: Additional file 2: — Figure of elemental profiles of the controls plants in LJ13 in μg.g−1 of leaf dry weight and their standard error bars. (PDF 20 kb) [file 12864_2016_3129_MOESM2_ESM.pdf]

Additional file 2

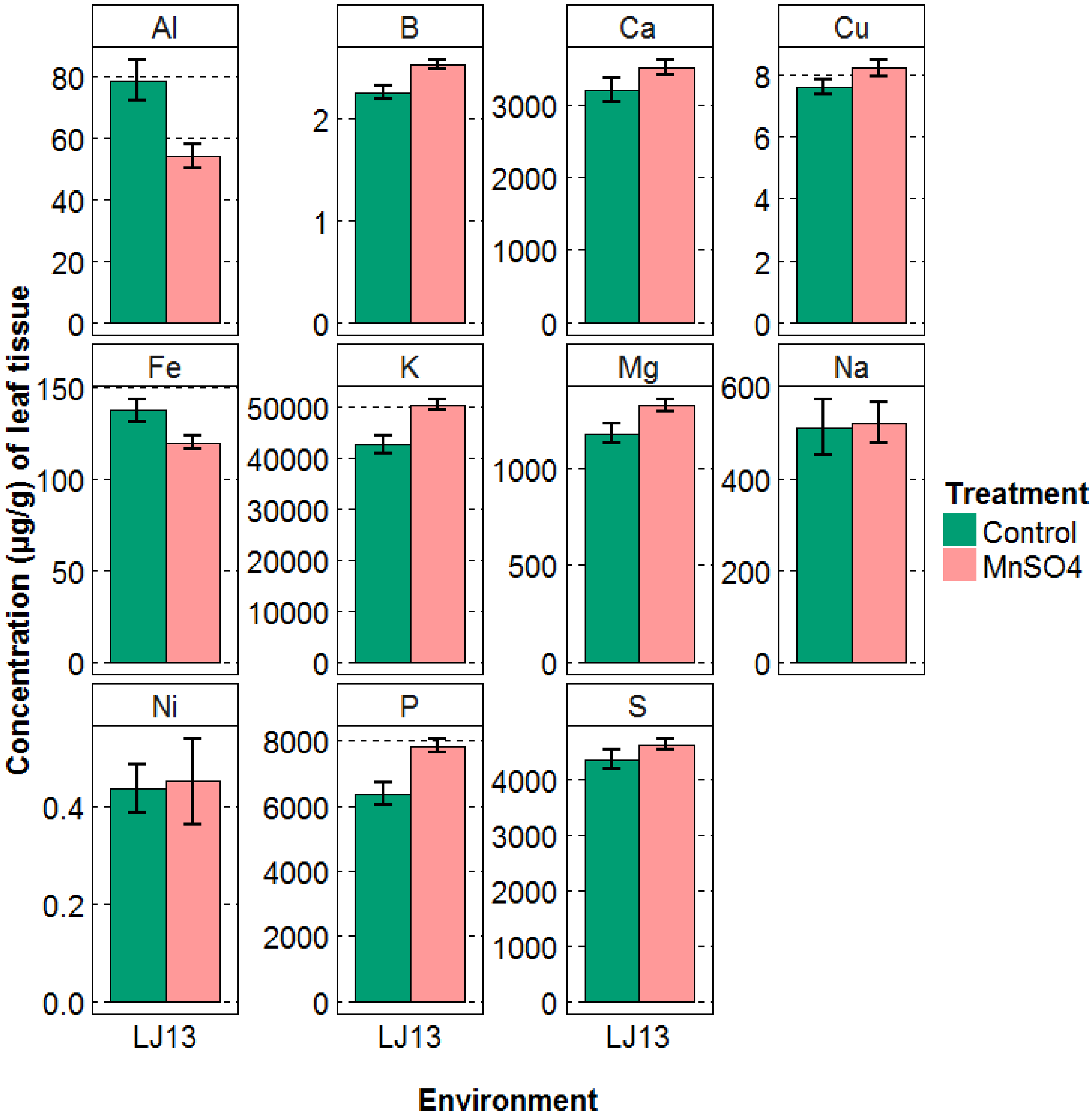

Figure of elemental profiles of the controls plants in LJ13 in µg.g^{-1} of leaf dry weight and their standard error bars.
