# Supplementary material for: Identification of manganese efficiency candidate genes in winter barley (Hordeum vulgare) using genome wide association mapping
Source: BMC Genomics. 2016 Oct 4;17:775. doi: 10.1186/s12864-016-3129-9 (PMC5050567; doi:10.1186/s12864-016-3129-9)
Supplement: Additional file 3: — Table of GWA results for Mn concentration. (PDF 13 kb) [file 12864_2016_3129_MOESM3_ESM.pdf]

**<u>Additional file 3</u>**

## Association results for Mn concentration

Summary of significant ($-\log_{10}(P_{Val}) \geq 3$) marker-trait associations identified by GWA for the Mn leaf concentration quantification and the corresponding candidate genes from their Blast results. Allelic effect sign are estimated with respect to the minor allele. SNPs are ordered by location and genome position. (*Minor Allele Frequency, ** Standard error).

| Location | Marker name | Position | | GWA statistics | | | | BLAST results | | |
|---|---|---|---|---|---|---|---|---|---|---|
| | | Chrom | cM | $-\log_{10}(P)$ | MAF* | Effect | SE** | Gene | cM | Description |
| GH13A | SCRI_RS_156295 | 5H | 31.8 | 3.1 | 0.28 | 0.031 | 0.009 | - | - | - |
| | 12_31044 | | 95.1 | 3.6 | 0.12 | 0.043 | 0.012 | - | - | - |
| | 12_31048 | 6H | 95.1 | 3.6 | 0.12 | 0.043 | 0.012 | | | |
| | SCRI_RS_6720 | | 104.8 | 3.0 | 0.16 | 0.034 | 0.010 | - | - | - |
