# Supplementary material for: Identification of manganese efficiency candidate genes in winter barley (Hordeum vulgare) using genome wide association mapping
Source: BMC Genomics. 2016 Oct 4;17:775. doi: 10.1186/s12864-016-3129-9 (PMC5050567; doi:10.1186/s12864-016-3129-9)
Supplement: Additional file 4: — Table of GWA results for JIP parameters. (PDF 49 kb) [file 12864_2016_3129_MOESM4_ESM.pdf]

Additional file 4

Association results for JIP parameters

Summary of significant ($-\log_{10}(P_{Val}) \geq 3$) marker-trait associations identified by GWA for the fluorescence parameters traits and the corresponding candidate genes from their Blast results. Allelic effect sign are estimated with respect to the minor allele. SNPs are ordered by location and genome position. (*Minor Allele Frequency, ** Standard error).

Fluorescence parameters	Location	Marker name	Position		GWA statistics				BLAST results		
			Chrom	cM	$-\log_{10}(P)$	MAF*	Effect	SE**	Gene	cM	Description
Area	GH13A	SCRI_RS_193456	5H	169.4	3.7	0.09	-3949	1038	-	-	-
		12_10596	6H	68.2	3.0	0.28	-2374	712	-	-	-
	GH13B	SCRI_RS_153727	1H	70.7	3.1	0.21	6386	1872	AK357833	70.7	Chlorophyll a/b binding protein
		SCRI_RS_1473		71.2	3.1	0.21	-6317	1873			
		SCRI_RS_169123		71.2	3.5	0.34	6103	1675			
		11_21057	2H	40.1	3.3	0.21	6493	1854	AK368803	40.0	Photosystem I PsaN, reaction centre subunit N
		11_20135	4H	54.3	3.2	0.08	-8619	2484	AK357955	54.3	PP2C, Mn/Mg aspartate binding site
		11_20472		54.3	3.2	0.08	8619	2484			
		11_21017		54.3	3.2	0.08	-8619	2484	AK249774.1	54.3	Photosystem II PsbP, oxygen evolving complex
		12_30839		54.3	3.2	0.08	8619	2484			
		11_20750	7H	61.5	4.5	0.42	-6357	1492	MLOC_56051.1	65.4	Chlorophyll a/b binding protein
		12_10657		61.5	4.5	0.42	-6357	1492			
		SCRI_RS_152299		61.7	3.9	0.33	-6237	1594			
		11_20975		61.8	4.8	0.40	-6569	1484			
		12_30149		62.4	4.2	0.36	6241	1531			
		SCRI_RS_132425		62.4	4.1	0.36	-6112	1512			
	LJ13	SCRI_RS_137263	2H	77.1	3.1	0.38	-4062	1196	AK364127	74.3	Photosystem II PsbQ, oxygen evolving complex
		11_10287		79.9	3.1	0.38	4062	1196			
		12_30897		80.0	3.9	0.47	-3326	859			

		12_30901		80.0	4.0	0.45	3355	847			
		SCRI_RS_196853		80.6	3.9	0.47	3326	859			
		SCRI_RS_88704		80.6	3.9	0.47	3326	859			
		SCRI_RS_126439		81.5	3.0	0.17	-4743	1416			
		SCRI_RS_159462		81.5	3.0	0.17	-4743	1416			
		SCRI_RS_160833		81.5	3.0	0.17	4743	1416			
		SCRI_RS_165463		81.5	3.0	0.17	-4743	1416			
		SCRI_RS_211281		81.5	3.0	0.17	-4743	1416			
		12_10936		81.8	3.7	0.24	3751	989			
		12_31424		81.8	3.7	0.24	3751	989			
		SCRI_RS_179213		81.8	3.0	0.17	-4743	1416			
		SCRI_RS_188339		81.8	3.0	0.17	-4743	1416			
		11_21351		82.5	3.7	0.24	3751	989			
		12_10969		82.5	3.7	0.24	3751	989			
		SCRI_RS_119261		82.5	3.7	0.24	3751	989			
		SCRI_RS_138463		82.5	3.0	0.17	-4743	1416			
		SCRI_RS_155456		82.5	3.6	0.25	-3661	990	MLOC_17228.1		
		SCRI_RS_162798		82.5	3.7	0.24	3751	989			
		SCRI_RS_183984		82.5	3.7	0.24	3751	989			
		SCRI_RS_237481		82.5	3.6	0.25	-3661	990			
		11_21037		82.8	4.7	0.40	-4943	1132			
		SCRI_RS_160616		86.9	3.0	0.17	4743	1416			
		SCRI_RS_235221		86.9	3.0	0.17	4743	1416			
		SCRI_RS_195051		146.5	3.0	0.41	2470	738	-	-	-
	**GH13A**	11_10294	1H	46.6	3.1	0.16	-0.008	0.002	MLOC_61942.1	50.9	PP2C, Mn/Mg aspartate binding site
		SCRI_RS_221759		46.6	3.9	0.08	-0.011	0.003			
		SCRI_RS_184488	7H	120.8	3.3	0.01	0.024	0.007	MLOC_77860.1<br>MLOC_3173.4	124.6<br>124.6	Photosystem II PsbP, oxygen evolving complex<br>PP2C, Mn/Mg aspartate binding site
	**GH13B**	12_30992		43.3	3.2	0.15	0.011	0.003			
		SCRI_RS_190764	4H	43.3	3.9	0.15	-0.012	0.003	-	-	-
		12_10371		43.5	3.9	0.15	-0.012	0.003			
		11_10834	5H	94.7	3.9	0.03	0.021	0.005	-	-	-
**V_I**	**LJ13**	11_20908	1H	116.5	3.1	0.40	0.004	0.001	MLOC_53469.2	117.5	Photosystem I PsaH, reaction centre subunit VI
		12_30823	2H	140.8	3.2	0.11	0.005	0.001	-	-	-
		SCRI_RS_209312		15.1	3.2	0.02	-0.011	0.003			
		SCRI_RS_135155	3H	135.5	3.0	0.03	0.008	0.002	-	-	-
		SCRI_RS_144313		135.5	4.4	0.01	0.015	0.003			
		SCRI_RS_238610	4H	69.0	3.4	0.01	0.013	0.004	-	-	-
		SCRI_RS_188572	5H	126.1	3.7	0.02	0.010	0.003	-	-	-
		SCRI_RS_2824		151.9	3.1	0.04	0.008	0.002	MLOC_73050.2	151.9	Photosystem I PsaE, reaction centre subunit IV
		SCRI_RS_158266	7H	12.8	3.1	0.36	-0.003	0.001	MLOC_69817.1	12.8	Mn/iron superoxide dismutase
		11_10294		46.6	4.0	0.16	-0.014	0.003			
		SCRI_RS_221759		46.6	5.9	0.08	-0.019	0.004			
		SCRI_RS_182631		46.8	5.6	0.04	-0.025	0.005			
		SCRI_RS_188937		46.8	5.6	0.04	-0.025	0.005			
**V_J**	**GH13A**	11_11287	1H	48.1	4.7	0.04	0.023	0.005	MLOC_61942.1	50.9	PP2C, Mn/Mg aspartate binding site
		11_20260		48.1	4.7	0.04	-0.023	0.005			
		11_20698		48.1	4.8	0.05	0.023	0.005			
		11_21193		48.1	4.7	0.04	-0.023	0.005			
		12_10300		48.1	4.7	0.04	-0.023	0.005			
		12_30043		48.1	4.8	0.05	0.023	0.005			

		12_30404		48.1	4.7	0.04	-0.023	0.005			
		12_30562		48.1	4.8	0.05	0.023	0.005			
		SCRI_RS_138691		48.2	5.1	0.04	0.026	0.006			
		11_21312		48.4	3.1	0.12	-0.011	0.003			
		11_21357		48.4	4.0	0.10	-0.015	0.004			
		SCRI_RS_171543		48.4	4.3	0.10	-0.015	0.004			
		SCRI_RS_7197		48.4	3.0	0.41	0.010	0.003			
		11_20912		48.7	3.1	0.12	-0.012	0.004			
		11_20432		60.1	3.1	0.10	0.012	0.004	-	-	-
		12_31099		61.2	3.4	0.09	0.014	0.004			
		12_11242		65.1	3.7	0.02	0.028	0.007	MLOC_56051.1	65.4	
		12_11045		67.8	5.2	0.02	0.037	0.008			
		12_30997	7H	67.8	4.0	0.02	0.028	0.007			Chlorophyll a/b binding protein
		12_11202		68.0	5.2	0.02	0.037	0.008	MLOC_20487.1	68.4	
		12_11103		68.1	5.2	0.02	-0.037	0.008			
		11_10526		46.5	3.1	0.46	-0.015	0.005			
		11_10294		46.6	3.1	0.16	-0.014	0.004			
		SCRI_RS_182631		46.8	3.7	0.04	-0.024	0.006			
		SCRI_RS_188937		46.8	3.7	0.04	-0.024	0.006			
		11_11287		48.1	3.1	0.04	0.023	0.007			
		11_20260		48.1	3.1	0.04	-0.023	0.007			
		11_21193		48.1	3.1	0.04	-0.023	0.007			
		12_10300	1H	48.1	3.1	0.04	-0.023	0.007	MLOC_61942.1	50.9	PP2C, Mn/Mg aspartate binding site
		12_30404		48.1	3.1	0.04	-0.023	0.007			
	**GH13B**	12_31208		48.1	4.1	0.40	0.014	0.004			
		SCRI_RS_124377		48.2	4.3	0.40	-0.015	0.004			
		SCRI_RS_138691		48.2	3.1	0.04	0.024	0.007			
		SCRI_RS_143927		48.2	4.1	0.40	-0.014	0.004			
		SCRI_RS_186830		48.2	4.5	0.41	0.015	0.004			
		SCRI_RS_189586		48.2	4.1	0.40	-0.014	0.004			
		SCRI_RS_7197		48.4	3.7	0.41	0.014	0.004			
		SCRI_RS_48964	2H	107.2	3.2	0.01	-0.042	0.012	-	-	-
		SCRI_RS_157504		120.7	3.0	0.02	0.028	0.008	AK373061	120.7	PP2C, Mn/Mg aspartate binding site
		11_20975	7H	61.8	3.1	0.40	0.010	0.003	MLOC_62559.1<br>MLOC_56051.1	56.3<br>65.4	Chlorophyll a/b binding protein
		12_30867	5H	126.1	3.0	0.19	0.003	0.001	-	-	-
		12_30868		126.1	3.9	0.09	-0.005	0.001			
		SCRI_RS_164602		53.3	3.0	0.37	0.004	0.001			
		SCRI_RS_210918		53.3	3.0	0.37	0.004	0.001	MLOC_72290.1	53.8	Chlorophyll a/b binding protein
		12_30857		56.2	3.2	0.49	-0.003	0.001			
	**LJ13**	11_11246		75.5	3.1	0.34	0.003	0.001			
		SCRI_RS_131992	6H	75.5	3.5	0.46	-0.003	0.001			
		SCRI_RS_144862		75.5	3.4	0.46	0.003	0.001			
		SCRI_RS_154582		75.5	3.1	0.47	0.003	0.001	MLOC_12843.2	78.3	Chlorophyll a/b binding protein
		SCRI_RS_181031		75.5	3.2	0.45	0.003	0.001			
		SCRI_RS_217187		75.5	3.1	0.47	-0.003	0.001			
		SCRI_RS_184811		76.1	3.1	0.34	-0.003	0.001			
		11_10526		46.5	3.2	0.46	-0.017	0.008			
**V_K**	**GH13A**	SCRI_RS_182631	1H	46.8	3.8	0.04	-0.034	0.011	MLOC_61942.1	50.85	PP2C, Mn/Mg aspartate binding site
		SCRI_RS_188937		46.8	3.8	0.04	-0.034	0.011			
		11_11287		48.1	3.7	0.04	0.038	0.012			

GH13B	11_20260	1H	48.1	3.7	0.04	-0.047	0.012	MLOC_61942.1	50.85	PP2C, Mn/Mg aspartate binding site
	11_20427		48.1	4.1	0.40	-0.026	0.006			
	11_20698		48.1	3.1	0.05	0.046	0.012			
	11_20698		48.1	3.9	0.05	0.046	0.012			
	11_21193		48.1	3.7	0.04	-0.047	0.012			
	12_10300		48.1	3.7	0.04	-0.047	0.012			
	12_30043		48.1	3.1	0.05	0.046	0.012			
	12_30043		48.1	3.9	0.05	0.046	0.012			
	12_30404		48.1	3.7	0.04	-0.047	0.012			
	12_30562		48.1	3.1	0.05	0.046	0.012			
	12_30562		48.1	3.9	0.05	0.046	0.012			
	12_31208		48.1	5.0	0.40	0.029	0.006			
	SCRI_RS_193640		48.1	4.1	0.40	-0.026	0.006			
	SCRI_RS_124377		48.2	5.2	0.40	-0.030	0.006			
	SCRI_RS_138691		48.2	3.8	0.04	0.050	0.013			
	SCRI_RS_143927		48.2	5.0	0.40	-0.029	0.006			
	SCRI_RS_186830		48.2	5.2	0.41	0.030	0.006			
	SCRI_RS_189586		48.2	5.0	0.40	-0.029	0.006			
	SCRI_RS_7197		48.4	4.2	0.41	0.026	0.006			
	11_20912		48.7	3.1	0.12	-0.017	0.008			
	SCRI_RS_48964	2H	107.2	3.7	0.01	-0.084	0.022	-	-	-
	SCRI_RS_157504		120.7	3.2	0.02	0.056	0.016	AK373061	120.68	PP2C, Mn/Mg aspartate binding site
LJ13	SCRI_RS_231015	2H	147.3	4.4	0.18	0.014	0.003	-	-	-
	11_21511	3H	52.6	3.7	0.35	-0.016	0.004	AK374059	52.62	PP2C, Mn/Mg aspartate binding site
	11_21414	5H	49.9	3.1	0.21	-0.010	0.003	AK368229<br>MLOC_18354.1	47.92<br>51.33	Chlorophyll a/b binding protein
	12_30826	7H	140.9	3.0	0.17	0.010	0.003	-	-	-
